# Teaghrelin Protects SH-SY5Y Cells against MPP^+^-Induced Neurotoxicity through Activation of AMPK/SIRT1/PGC-1α and ERK1/2 Pathways

**DOI:** 10.3390/nu12123665

**Published:** 2020-11-28

**Authors:** Cian-Fen Jhuo, Sheng-Kuo Hsieh, Chun-Jung Chen, Wen-Ying Chen, Jason T.C. Tzen

**Affiliations:** 1 Graduate Institute of Biotechnology, National Chung Hsing University, Taichung 402, Taiwan; quartz1248s@gmail.com (C.-F.J.); vincent760424@yahoo.com.tw (S.-K.H.); 2 Department of Medical Research, Taichung Veterans General Hospital, Taichung 40705, Taiwan; cjchen@vghtc.gov.tw; 3 Department of Veterinary Medicine, National Chung Hsing University, Taichung 402, Taiwan

**Keywords:** Parkinson’s disease, neurodegeneration, teaghrelin

## Abstract

The prevalence and incidence of Parkinson’s disease (PD), an age-related neurodegenerative disease, are higher among elderly people. Independent of etiology, dysfunction and loss of dopaminergic neurons are common pathophysiological changes in PD patients with impaired motor and non-motor function. Currently, preventive or therapeutic treatment for combating PD is limited. The ghrelin axis and ghrelin receptor have been implicated in the preservation of dopaminergic neurons and have potential implications in PD treatment. Teaghrelin, a compound originating from Chin-Shin Oolong tea, exhibits ghrelin agonist activity. In this study, the neuroprotective potential of teaghrelin against PD was explored in a cell model in which human neuroblastoma SH-SY5Y cells were treated with the mitochondrial toxin 1-methyl-4-phenylpyridinium (MPP^+^). Upon MPP^+^ exposure, SH-SY5Y cells exhibited decreased mitochondrial complex I activity and apoptotic cell death. Teaghrelin activated AMP-activated protein kinase (AMPK)/sirtuin 1(SIRT1)/peroxisome proliferator-activated receptor gamma (PPARγ) coactivator-1α (PGC-1α) and extracellular signal–regulated kinases 1 and 2 (ERK1/2) pathways to antagonize MPP^+^-induced cell death. Herein, we propose that teaghrelin is a potential candidate for the therapeutic treatment of PD.

## 1. Introduction

Parkinson’s disease (PD) is a progressive movement disorder resulting from the gradual loss of dopaminergic neurons in substantia nigra pars compacta (SNpc) projecting into the striatum; this connecting pathway is known as the nigrostriatal pathway. With the reduction of dopamine (DA) levels in the striatum, patients with PD exhibit motor and non-motor dysfunction, including rigidity, bradykinesia, and depression [1]. According to an epidemiologic study, PD mainly occurs in people over the age of 65 years, with an incidence rate of 1–2% [2]. Currently, the most common treatment for PD is levodopa, which effectively improves clinical symptoms. However, several motor complications of levodopa are challenging to treat [3,4]. Levodopa does not enable the recovery of losses in dopaminergic neurons and does not delay or protect against progression of the disease; thus, the development of new adjuvant therapies is crucial.

Ghrelin, a 28-amino-acid orexigenic peptide hormone, stimulates growth hormone secretion by binding to growth hormone secretagogue receptor 1a (GHS-R1a). Because both ghrelin and its receptor are widely expressed in multiple regions of the brain [5,6], ghrelin and its signaling play crucial roles in the development of many diseases, such as anorexia, muscle atrophy, cancer, and neurodegenerative diseases [7,8]. Moreover, a recent study reported that the ghrelin agonist HM01 effectively attenuated non-motor dysfunction in a PD rat model [9]. Thus, the diverse effects of GHS-R1a and ghrelin establish them as promising drug development targets [9,10].

In modern society, tea drinking is regarded as a healthy behavior. Tea contains abundant bioactive ingredients that have effects that contribute to the prevention and treatment of age-related diseases, such as cancers, heart diseases, and neurodegenerative diseases [11]. Additionally, the daily consumption of tea reduces the risk of age-related brain decline [12]. Teaghrelin (TG) is an acylated flavonoid tetraglycoside extracted from Chin-Shin Oolong tea, a widely planted tea cultivar in Taiwan. In other studies, we demonstrated the efficacy of teaghelin in enhancing growth hormone secretion from primary anterior pituitary cells, ameliorating muscle atrophy in C2C12 myoblast cells, and stimulating the appetite of rats [13,14]. Research involving molecular docking has also demonstrated sufficient binding and interaction between teaghrelin and GHS-R1a [15]. These results indicate a potential similarity in the biological functions of teaghrelin to those of ghrelin. In addition, research has demonstrated the neuroprotection ability of ghrelin in patients with PD [16,17]. MPP^+^ is a potent neurotoxin extensively used to model PD. In this study, we investigated whether teaghrelin possesses neuroprotective effects in an MPP^+^-induced cell model of PD and the possible mechanism underlying these effects.

## 2. Materials and Methods

### 2.1. Materials

Cells were cultured in MEM /F12 (1:1 mixture) supplemented with fetal bovine serum (FBS), sodium pyruvate, and penicillin/streptomycin (Gibco, Waltham, MA, USA). PGC-1α, and β-actin were purchased from Novus Biologicals (Littleton, CO, USA), and Merck Millipore (Danvers, MA, USA), respectively; other antibodies were purchased from Cell Signaling (Danvers, MA, USA). The acylated ghrelin (purity: >95%) was purchased from Karebay Biochem (Monmouth Junction, NJ, USA). All chemicals were obtained from Sigma-Aldrich (St. Louis, MO, USA), unless otherwise indicated.

### 2.2. Preparation of Teaghrelin

Teaghrelin was isolated from Chin-Shin Oolong tea purchased from local tea producers and was extracted following a previous elution method with modification [18]. In brief, the extraction of tea was performed through column chromatography using Diaion HP-20 gel (Merck Millipore, Burlington, MA, USA) eluted with a 25%, 50%, and 75% methanol aqueous solution. The 75% ethanol elution was collected and further purified on Sephadex LH-20 gel (Merck Millipore, Burlington, MA, USA) eluted with a 30%, and 60% methanol aqueous solution. Finally, the third fraction (60%) was collected and analyzed using high-performance liquid chromatography (Waters Corporation, Milford, MA, USA).

### 2.3. Cell Culture and Differentiation

The SH-SY5Y human neuroblastoma cell line was purchased from ATCC (Manassas, VA, USA). Cells were grown in MEM/F12 (1:1 mixture) supplemented with 10% FBS, 1 mM sodium pyruvate, 2.2 g/L sodium bicarbonate, and 1% penicillin/streptomycin in a 5% CO_2_ incubator at 37 °C. For inducing the differentiation of SH-SY5Y cells into dopaminergic cells, SH-SY5Y cells were cultured for 3 days in MEM/F12 medium containing 10 μM retinoic acid and 3% FBS, followed by culturing for a further 3 days with 80 nM phorbol 12-myristate 13-acetate in MEM/F12 medium containing 3% FBS. All media were supplemented with 1 mM sodium pyruvate, 2.2 g/L sodium bicarbonate, and 1% penicillin/streptomycin. Cells were maintained at 37 °C in a humidified atmosphere of 5% CO_2_.

### 2.4. Cell Viability Assay

Cell viability was determined through 2,3-bis (2-methoxy-4-nitro-5-sulfophenyl)-2H-tetrazolium-5-carboxanilide (XTT) assay (Biological Industries, Biological Industries, Cromwell CT, USA.) SH-SY5Y cells were grown in 48-well plates to a confluence of 1 × 10^4^ cells/mL. They were then treated with or without MPP^+^ (1, 2, and 3 mM), teaghrelin (1, 10, and 100 μM), ghrelin (positive control, 1 μM), and [D-Arg(1),D-Phe(5),D-Trp(7,9),Leu(11)]-substance P (ghrelin receptor antagonist, 0.5 μM). The doses of ghrelin and MPP^+^ were the same as those used in previous studies [19,20], and teaghrelin was modified with ghrelin. After 24-h treatment, an XTT solution was added to the wells in accordance with manufacturer’s instruction, with incubation at 37 °C for 4 h. Absorbance was measured at 540 nm using a Multiskan GO Microplate Spectrophotometer (Thermo-Fisher Scientific, Waltham, MA, USA).

### 2.5. Immunofluorescence Staining

SH-SY5Y cells were seeded at 7 × 10^5^ cells/mL and differentiated on poly-D-lysine/laminin–treated coverslips, which were placed in a 12-well plate. Cells were treated with or without ghrelin, teaghrelin, and MPP^+^ for 24 h. The next day, cells were washed with phosphate-buffered saline (PBS) three times and immediately fixed with 4% paraformaldehyde for 1 h at room temperature. After cells were permeabilized for 20 min in 0.3% Triton-100, they were blocked with 1% BSA for 1 h and incubated with primary antibody against TH at 4 °C overnight. The next day, cells were washed three times with PBS and then incubated at room temperature for 1 h with anti-rabbit IgG Alexa 488 secondary antibody. Coverslips were mounted with fluoroshield medium containing DAPI (Abcam, Cambridge, MA, USA), and cells were evaluated using a fluorescence microscope (IX71; Olympus, FL, USA).

### 2.6. Measurement of Mitochondrial Complex I Activity

After various treatments, mitochondrial complex I activity was measured using a Complex I Enzyme Activity Microplate Assay Kit (Abcam, Cambridge, MA, USA) in accordance with the manufacturer’s instructions.

### 2.7. Western Blotting

SH-SY5Y cells with various treatments were lysed in M-PER reagent (Thermo-Fisher Scientific, Waltham, MA, USA) and 1% phosphatase and protease inhibitor cocktails (Merck Millipore, Burlington, MA, USA). Cells were immediately harvested and centrifuged at 12,000 rpm for 15 min at 4 °C. The supernatants were collected, and the protein concentration was assessed using Bradford protein assay (Bio-Rad Laboratories, Irvine, CA, USA). Protein samples (50 μg) were resolved through 10% or 12.5% sodium dodecyl sulfate-polyacrylamide gel electrophoresis (SDS-PAGE) were then transferred onto 0.22-μm polyvinylidene fluoride membranes (Merck Millipore, Burlington, MA, USA). After the membranes were immersed in BlockPRO™ blocking buffer (Energenesis Biomedical, Taipei, Taiwan) at room temperature for 1 h, they were incubated with primary antibodies, diluted with blocking buffer, against PINK1, cytochrome c, caspase-3, TH, AMPK, p-AMPK, SIRT1, PGC-1α, ERK1/2, p-ERK1/3, and β-actin overnight at 4 °C. The next day, the membranes were washed three times with PBS and then incubated with the corresponding secondary antibodies for 1 h at room temperature. After several washes with PBS proteins were detected using an enhanced chemiluminescence reagent (Thermo-Fisher Scientific, Waltham, MA, USA) with an imaging system and quantified using ImageJ software (National Institutes of Health, Stapleton, NY, USA).

### 2.8. Statistical Analysis

Data are presented as the mean ± standard deviation. All statistical analyses were performed using one-way analysis of variance followed by Duncan’s post hoc multiple comparison test. Statistics were calculated using SigmaPlot 12.0 software (Systat, Chicago, IL, USA), and *p* < 0.05 was considered statistically significant.

## 3. Results

### 3.1. Teaghrelin Attenuated MPP^+^-Induced Cytotoxicity

**Figure 1:**
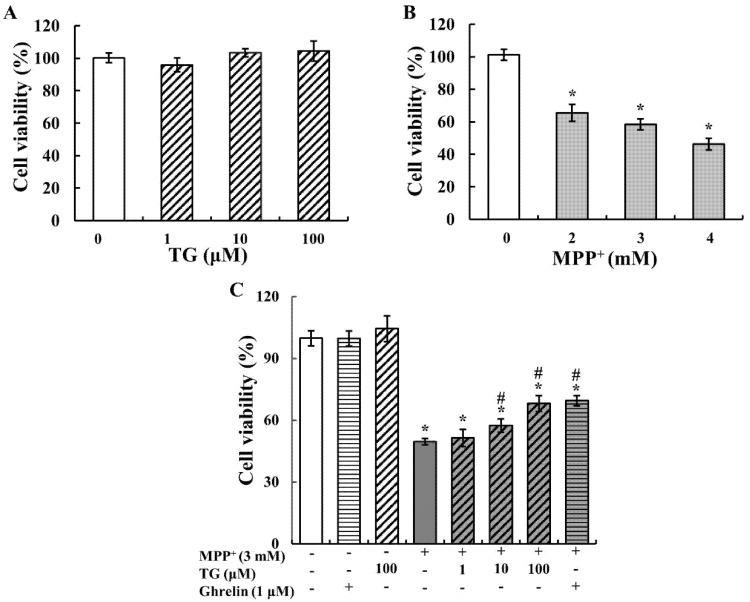
Teaghrelin (TG) protected cells against 1-methyl-4-phenylpyridinium (MPP^+^)-induced cytotoxicity. Cells were treated with different concentrations of teaghrelin or MPP^+^ for 24 h ((**A**,**B**) respectively). Cells were treated with or without TG, ghrelin, and MPP^+^ for 24 h (**C**). Cell viability was measured using 2,3-bis (2-methoxy-4-nitro-5-sulfophenyl)-2H-tetrazolium-5-carboxanilide (XTT) assay. The results were analyzed using one-way analysis of variance. Values are presented as the mean ± standard deviation (*n* = 4). * *p* < 0.05, compared with the control group, ^#^ *p* < 0.05 compared with MPP^+^ alone. -: untreated with drugs or compounds, +: treated with drugs or compounds.

To determine the optimal dose for the experiment, SH-SY5Y cells were exposed to different concentrations of teaghrelin and MPP^+^ for 24 h, and cell viability was measured using 2,3-bis (2-methoxy-4-nitro-5-sulfophenyl)-2H-tetrazolium-5-carboxanilide (XTT) assay. As shown in Figure 1A, teaghrelin did not exert any significant effect on cell viability. This finding suggests that teaghrelin is safe to use and has no toxicity for SH-SY5Y cells. MPP^+^ is widely used as a PD-related neurotoxin. As shown in Figure 1B, MPP^+^ significantly reduced neural cell viability. The optimal dose was 3 mM MPP^+^, which reduced cell viability to 50–60%, and was thus used in subsequent experiments. To evaluate the protective effects of teaghrelin on MPP^+^-induced cell death, cells were treated with teaghrelin (1, 10, and 100 μM) or ghrelin (1 μM) in the presence or absence of MPP^+^ (3 mM) for 24 h. The results showed that teaghrelin of 10 and 100 μM and ghrelin of 1 μM significantly attenuated MPP^+^-induced cytotoxicity (Figure 1C). These findings suggest that teaghrelin exerts protective effects against MPP^+^-induced cytotoxicity.

### 3.2. Teaghrelin Alleviated Mitochondrial Dysfunction and Apoptosis in the MPP^+^-Induced SH-SY5Y Cell Model of PD

**Figure 2:**
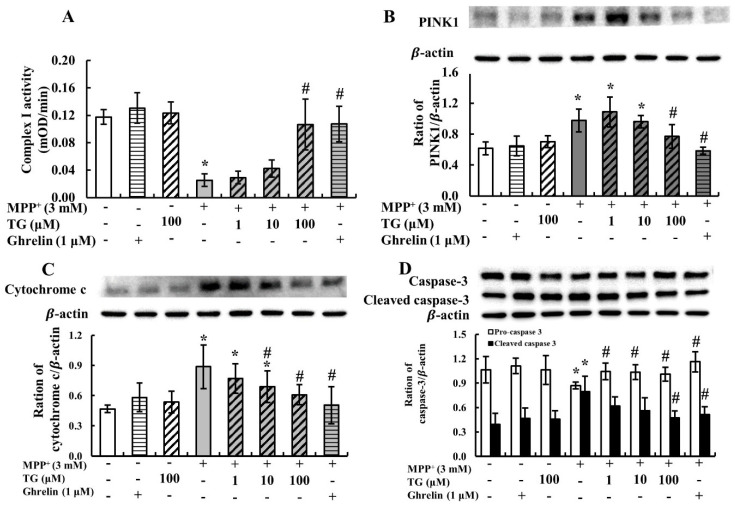
Teaghrelin (TG) attenuated 1-methyl-4-phenylpyridinium (MPP^+^)-induced mitochondrial dysfunction and apoptosis in SH-SY5Y cells. SH-SY5Y cells were treated with TG or ghrelin in the presence or absence of MPP^+^ for 24 h. After various treatments, mitochondrial complex I activity was measured at 450 nm using a microplate reader (**A**). Cells were harvested and analyzed through Western blotting using antibodies against phosphatase and tensin homologue-induced kinase 1 (PINK1) (**B**), cytochrome c (**C**), and caspase-3 (**D**). The expression of β-actin was used as the internal control. Results were analyzed with one-way analysis of variance. Values are presented as the mean ± standard deviation (*n* = 3). * *p* < 0.05 compared with the control group, ^#^ *p* < 0.05 compared with MPP ^+^ alone. -: untreated with drugs or compounds, +: treated with drugs or compounds.

MPP^+^ has been widely used in PD models for the inhibition of mitochondrial complex I. Thus, in this study, we measured mitochondrial complex I activity in SH-SY5Y cells treated with MPP^+^ and with or without teaghrelin (100 μM). As presented in Figure 2A, MPP^+^ significantly reduced the enzymatic activity of mitochondrial complex I, and the reduction was effectively reversed by teaghrelin. These results indicate that teaghrelin can alleviate MPP^+^-induced cytotoxicity by preserving mitochondrial complex I activity.

Phosphatase and tensin homologue-induced kinase 1 (PINK1) is a mitochondrial kinase affecting mitochondrial health, and it promotes cell survival. Growing evidence indicates that PINK1-mediated mitophagy is involved in the development of PD. When mitochondria are damaged, PINK1 accumulates on the outer mitochondrial membrane (OMM), leading to mitophagy for maintaining cell function. In this study, to further investigate whether teaghrelin exerts a neuroprotective effect by modulating PINK1-mediated mitophagy, PINK1 expression was measured using Western blotting. As shown in Figure 2B, MPP^+^ significantly increased PINK1 expression. However, in comparison with the MPP^+^ group, treatment with teaghrelin (100 μM) or ghrelin (1 μM) significantly reduced the expression of PINK1 in MPP^+^-induced cells. These observations indicate that teaghrelin can protect neuronal cells by alleviating MPP^+^-induced mitochondrial dysfunction.

Mitochondrial dysfunction due to MPP^+^ exposure may result in apoptosis and induce neuronal cell death. Once mitochondria are damaged, cytochrome c is released from mitochondria and activates the downstream effector caspase-3, which in turn mediates apoptosis. In this study, to confirm whether teaghrelin protects cells from apoptosis in the PD model, cytochrome c and caspase-3 levels were measured using Western blotting. Compared with the control, MPP^+^ reduced the procaspase-3 level but enhanced cytochrome c and cleaved caspase-3 levels. Furthermore, teaghrelin (100 μM) significantly reduced the expression of cytochrome c and cleaved caspase-3 in the PD model (Figure 2C,D). Thus, teaghrelin treatment attenuated MPP^+^-induced apoptosis.

### 3.3. Teaghrelin Attenuated MPP^+^-Induced Loss of Tyrosine Hydroxylase Expression in SH-SY5Y Cells

**Figure 3:**
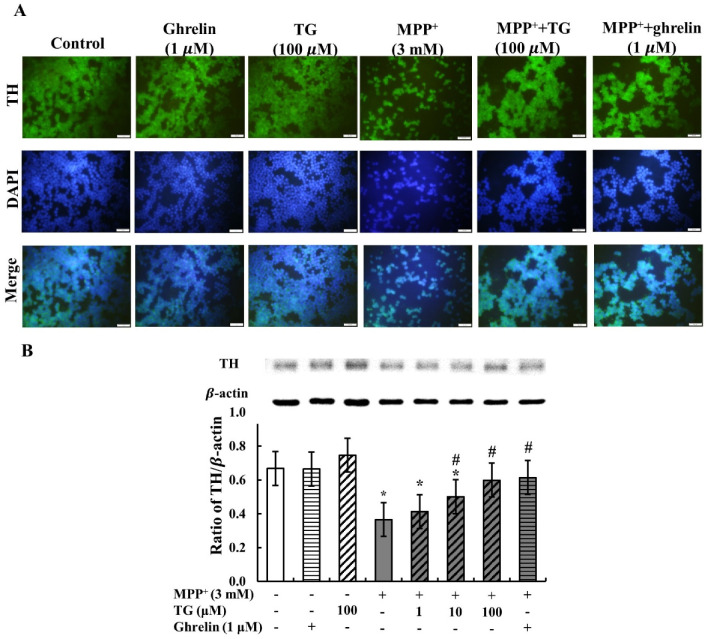
Teaghrelin (TG) attenuated 1-methyl-4-phenylpyridinium (MPP^+^)-induced loss of tyrosine hydroxylase (TH) expression in SH-SY5Y cells. (**A**) Immunofluorescence staining of tyrosine hydroxylase (green) and DAPI (blue) in each group of SH-SY5Y cells with various treatments (scale bar, 50 µm). (**B**) SH-SY5Y cells treated with or without TG, ghrelin, and MPP^+^ for 24 h. The expression of TH was analyzed through Western blotting. The expression of β-actin was used as the internal control. The results were analyzed through one-way analysis of variance. Values are presented as the mean ± standard deviation (*n* = 3). * *p* < 0.05 compared with the control group, ^#^ *p* < 0.05 compared with MPP ^+^ alone. -: untreated with drugs or compounds, +: treated with drugs or compounds.

PD is pathologically characterized by the loss of dopaminergic neurons and the depletion of DA in SNpc. Tyrosine hydroxylase (TH) is the rate-limiting enzyme in DA biosynthesis. Reduction of TH expression results in diminished DA synthesis and leads to PD. Thus, TH plays a vital role in the pathogenesis of PD. In this study, immunofluorescence was used to assess TH expression to investigate whether it is protected by teaghrelin in the PD cell model. Cell nuclei staining and TH assay revealed that MPP^+^ treatment reduced not only cell viability but also TH expression. However, cell viability and TH expression were increased in SH-SY5Y cells treated with teaghrelin and ghrelin compared with MPP^+^-induced cells without any treatment (Figure 3A).

To further determine whether teaghrelin directly affects TH expression in MPP^+^-induced SH-SY5Y cells, TH levels were measured using Western blotting. As shown in Figure 3B, TH expression was decreased in MPP^+^-induced SH-SY5Y cells compared with control cells. Teaghrelin or ghrelin alone did not have any effect on TH expression. In cells co-treated with MPP^+^, teaghrelin or ghrelin effectively recovered TH expression. Thus, we suggest that teaghrelin attenuates MPP^+^-induced loss of TH expression in SH-SY5Y cells.

### 3.4. Substance P Attenuated the Protective Effect of Teaghrelin on MPP^+^-Induced Neurotoxicity in SH-SY5Y Cells

**Figure 4:**
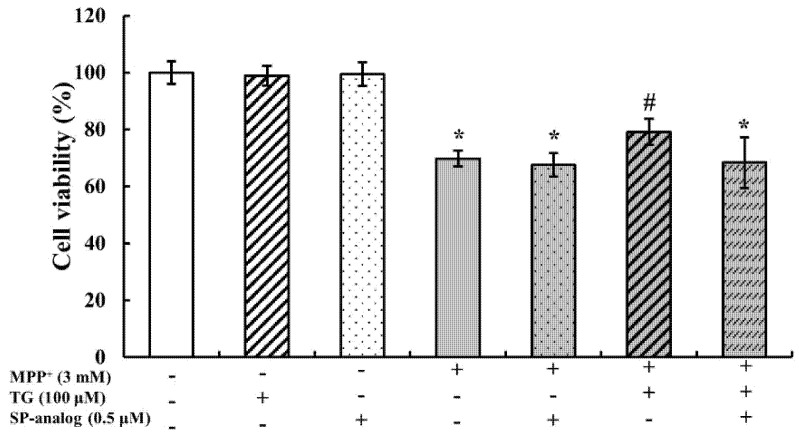
Substance P (SP-analog) attenuated the protective effects of teaghrelin (TG) on 1-methyl-4-phenylpyridinium (MPP^+^)-induced cytotoxicity. SH-SY5Y cells were treated with TG or SP-analog in the presence or absence of MPP^+^ for 24 h. Cell viability was measured using2,3-bis (2-methoxy-4-nitro-5-sulfophenyl)-2H-tetrazolium-5-carboxanilide (XTT) assay. The results were analyzed using one-way analysis of variance. Values are presented as the mean ± standard deviation (*n* = 4). * *p* < 0.05 compared with the control group, ^#^ *p* < 0.05 compared with MPP^+^ alone. -: untreated with drugs or compounds, +: treated with drugs or compounds.

As mentioned, teaghrelin could protect SH-SY5Y cells from MPP^+^-induced neurotoxicity. Next, we explored the possibly neuroprotective pathway of teaghrelin. Based on our previous findings from primary anterior pituitary cells and molecular docking [13,15], a potential similarity in the biological functions between teaghrelin and ghrelin is hypothesized. Thus, we speculate that teaghrelin alleviates MPP^+^-induced neurotoxicity partially through GHS-R1a pathway. Evidence indicates that undifferentiated SH-SY5Y cells express the GHS-R1a receptor [21] and differentiated SH-SY5Y cells express the G protein-coupled receptors (GPCRs) [22,23]. [D-Arg(1),D-Phe(5),D-Trp(7,9),Leu(11)]-substance P (SP analog) is a relatively broad-spectrum antagonist of GPCRs [24], and a ghrelin antagonist [25]. Thus, we blocked the ghrelin receptor using SP analog (0.5 μM); the dose was the same as that used in previous studies [13,26]. Cells were pretreated with the GHS-R1a antagonist SP analog for 30 min and were then incubated in Minimum Essential Medium (MEM)/Ham’s F-12 Nutrient Mixture (F12) with teaghrelin (100 μM) or MPP^+^ (3 mM) for 24 h. When ghrelin receptors were blocked by the antagonist, cell survival decreased. However, with MPP^+^ and teaghrelin cotreatment without the ghrelin antagonist, teaghrelin could protect cells from MPP^+^-induced cytotoxicity (Figure 4). This result suggests that teaghrelin alleviates MPP^+^-induced neurotoxicity possibly through the ghrelin receptor or GPCRs.

**Figure 5:**
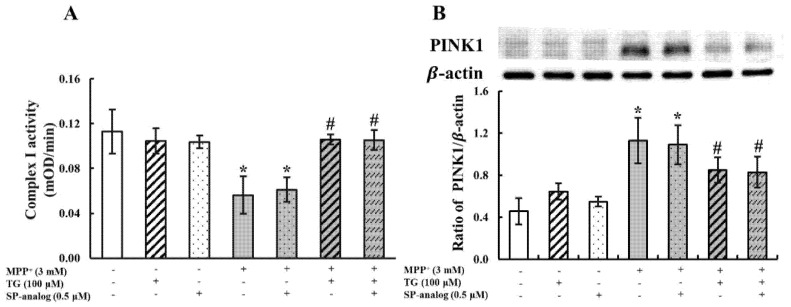
The protective effects of teaghrelin (TG) on 1-methyl-4-phenylpyridinium (MPP^+^)-induced mitochondrial dysfunction was not blocked by substance P (SP-analog). SH-SY5Y cells were treated with TG or SP analog in the presence or absence of MPP^+^ for 24 h. After various treatments, mitochondrial complex I activity was measured at 450 nm using a microplate reader (**A**). Cells were harvested and analyzed through Western blotting using antibodies against phosphatase and tensin homologue-induced kinase 1 (PINK1) (**B**). The expression of β-actin was used as the internal control. The results were analyzed using one-way analysis of variance. Values are presented as the mean ± standard deviation (*n* = 3). * *p* < 0.05 compared with the control group, ^#^ *p* < 0.05 compared with MPP^+^ alone. -: untreated with drugs or compounds, +: treated with drugs or compounds.

In the MPP^+^-induced PD model, mitochondrial complex I activity was inhibited, and apoptosis subsequently occurred. To investigate whether teaghrelin attenuates MPP^+^-induced mitochondrial dysfunction through GHS-R1a or GPCRs, we measured mitochondrial complex I activity and PINK1 expression in SH-SY5Y cells treated with MPP^+^ and teaghrelin with or without the substance P. As shown in Figure 5A, mitochondrial complex I activity was dramatically decreased after MPP^+^ treatment. However, teaghrelin treatment with or without the substance P attenuated the effect of MPP^+^ and prevented a reduction in complex I activity. By contrast, PINK1 expression increased after MPP^+^ treatment. Teaghrelin treatment with or without the substance P alleviated the effect of MPP^+^ and prevented the enhancement of PINK1 expression (Figure 5B). These results suggest that teaghrelin attenuates MPP^+^-induced mitochondrial dysfunction but possibly not through the ghrelin receptor or GPCRs.

**Figure 6:**
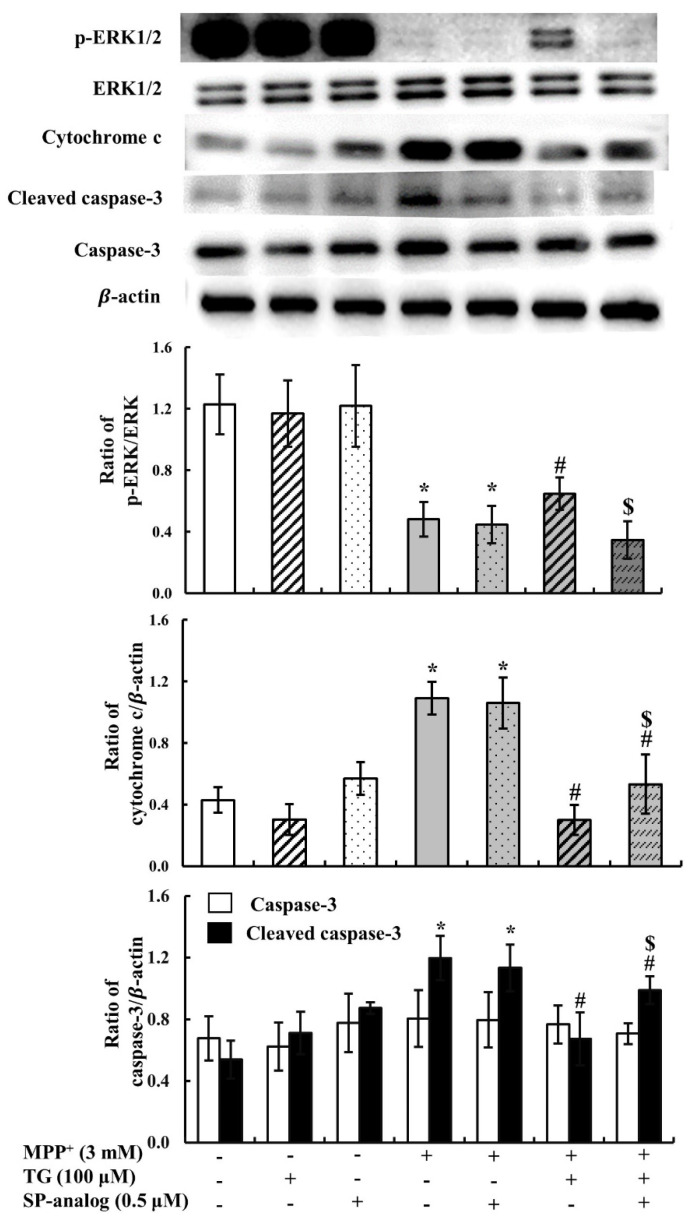
Teaghrelin (TG) attenuated 1-methyl-4-phenylpyridinium (MPP^+^)-induced neurotoxicity through activation of the extracellular signal-regulated kinase 1 and 2 (ERK1/2) pathway. SH-SY5Y cells were treated with TG or substance P (SP-analog) in the presence or absence of MPP^+^ for 24 h. Cells were harvested and analyzed through Western blotting using antibodies against ERK1/2, p-ERK1/2, cytochrome c, caspase-3, and cleaved caspase-3. The expression of β-actin and was used as the internal control. Values are presented as the mean ± standard deviation (n = 4). * *p* < 0.05 compared with the control group, ^#^ *p* < 0.05 compared with MPP^+^ alone. ^$^ *p* < 0.05 compared with the MPP^+^ + TG group. -: untreated with drugs or compounds, +: treated with drugs or compounds.

In addition to improving mitochondrial function, reduction of apoptosis is another mechanism of neuroprotection. One study showed that ghrelin activated ERK1/2 against MPP^+^-induced apoptosis [27]. In the present study, to further evaluate whether the anti-apoptotic effects of teaghrelin are mediated through the activation of the ERK1/2 pathway, the PD cell model was incubated with the substance P. As shown in Figure 6, ERK1/2 phosphorylation dramatically decreased, and cytochrome c and cleaved caspase-3 levels significantly increased after MPP^+^ treatment. Teaghrelin improved the effect of MPP^+^, but teaghrelin and substance P treatment mitigated the protective effect of teaghrelin. These results suggested that teaghrelin attenuates MPP^+^-induced apoptosis through activation of the GHS-R1a or GPCRs mediated ERK1/2 pathway.

**Figure 7:**
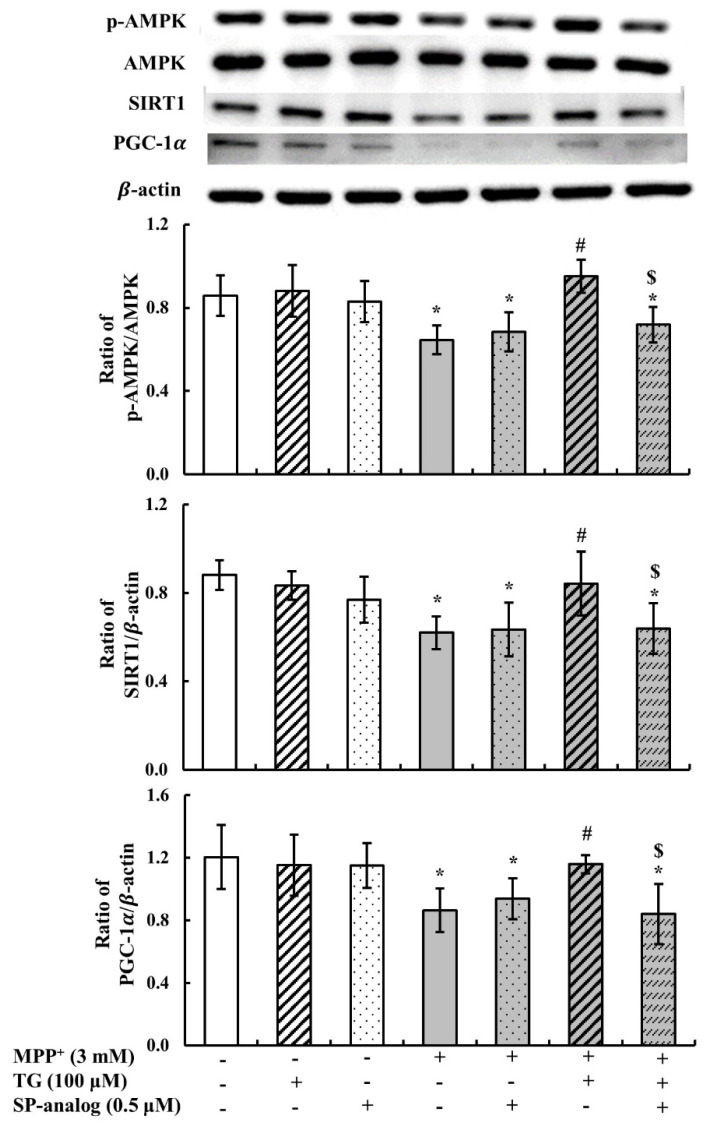
Teaghrelin (TG) attenuated 1-methyl-4-phenylpyridinium (MPP^+^)-induced neurotoxicity through activated AMP-activated protein kinase (AMPK)/sirtuin 1(SIRT1)/peroxisome proliferator-activated receptor gamma (PPARγ) coactivator-1α (PGC-1α). SH-SY5Y cells were treated with TG, SP-analog, respectively, in the presence or absence MPP^+^ for 24 h. Cells were harvested and determined by Western blotting using antibodies against AMPK, p-AMPK, SIRT, PGC-1α. The expression of β-actin was used as internal control. Values are presented as mean ± SD (n = 4). * *p* < 0.05 compared with control group, # *p* < 0.05 compared with MPP + alone. ^$^ *p* < 0.05 compared with MPP^+^+TG group. -: untreated with drugs or compounds, +: treated with drugs or compounds.

Ghrelin can promote mitochondrial biogenesis by activating the AMPK/SIRT1/PGC1-α pathway, depending on its ability to bind to GHS-R1a [28]. In this study, to further confirm whether the protective effect of teaghrelin in the PD cell model was mediated through activation of the AMPK/SIRT1/PGC1-α pathway, SH-SY5Y cells were incubated with the substance P. As shown in Figure 7, AMPK phosphorylation, SIRT1 expression, and PGC1-α expression were significantly decreased after MPP^+^ treatment. Teaghrelin attenuated the effect of MPP^+^, but teaghrelin and substance P treatment blocked the protective effect of teaghrelin. These findings suggest that teaghrelin can activate the AMPK/SIRT1/PGC1-α pathway through the ghrelin receptor or GPCRs.

## 4. Discussion

The cause of PD remains unclear, and PD is difficult to diagnose accurately. In general, at the onset of PD symptoms, approximately 30% of DA neurons are lost [29]. Currently, various therapeutic strategies for PD focus on DA replacement [1]. Another therapeutic strategy is neuroprotection, which involves protection against dopaminergic neuron death and loss. A previous study found that levels of GHS-R1a, the only functional ghrelin receptor, were dramatically decreased in PD-specific induced pluripotent stem cell-derived dopaminergic neurons [30]. A recent study also showed that the ghrelin agonist effectively attenuated non-motor dysfunction in a PD rat model [9]. Therefore, the ghrelin axis and ghrelin receptor have been suggested to be involved in the preservation of dopaminergic neurons, with potential implications in PD treatment. Teaghrelin, a compound extracted from Chin-Shin Oolong tea, exhibits ghrelin agonist activity. The results of the present study demonstrate that teaghrelin treatment reduced mitochondrial damage and apoptotic cell death caused by MPP^+^ exposure in SH-SY5Y cells, as evidenced by enhanced cell viability and TH expression; the treatment also attenuated complex I activity and PINK1 expression and inhibited cytochrome c release and caspase-3 activity. A previous study showed that ghrelin promoted mitochondrial biogenesis and inhibited apoptosis by activating AMPK/SIRT1/PGC1-α and ERK1/2 pathways, respectively. Expression levels of SIRT1 and PGC1-α and phosphorylation levels of AMPK and ERK1/2 were significantly reduced after MPP^+^ exposure, and treatment of cells with teaghrelin significantly prevented MPP^+^-induced reduction in expression and phosphorylation. However, the protective effects of teaghrelin against MPP^+^-induced neurotoxicity were blocked by substance P, a relatively broad-spectrum antagonist of GPCRs and a ghrelin antagonist, through the activation of AMPK/SIRT1/PGC-1α and ERK1/2 pathways. The current findings reveal that teaghrelin can be developed into a functional food, with the aim of preventing and treating PD.

Many studies have proposed that mitochondrial dysfunction has an integral role in the development of PD [31]. MPP^+^ specifically interferes with mitochondrial complex I activity and has been shown to induce mitochondrial dysfunction, causing electron transport chain activity deficiency and increasing mitophagy and apoptosis in the substantia nigra [32,33]. A previous study showed that ghrelin promotes cell survival by improving mitochondrial dysfunction [34]. In the present study, MPP^+^ treatment significantly reduced mitochondrial complex I activity. However, teaghrelin treatment with or without the substance P attenuated the effect of MPP^+^ and prevented a reduction in complex I activity. The expression of PINK1, a PD-associated protein involved in mitochondrial quality control, is increased in cases of mitochondrial dysfunction. In healthy mitochondria, PINK1 can be imported into the mitochondria inner membrane and then cleaved by presenilins-associated rhomboid-like protein. Dysfunctional mitochondria fail to degrade PINK1; thus, PINK1 is accumulated on the OMM. Thereafter, PINK1 recruits parkin to activate downstream signaling, and mitophagy is initiated for maintaining cell function [35]. In the present study, we found that PINK1 expression was increased after MPP^+^ treatment. Teaghrelin treatment with or without the substance P alleviated the effect of MPP^+^ and prevented the enhancement of PINK1 expression. Taken together, these results suggest that teaghrelin can attenuate mitochondrial complex I deficiency through another unknown pathway instead of binding to the ghrelin receptor or GPCRs. It has been reported that the effects of ghrelin on neural cells are mainly dependent of GHS-R1a receptor [36]. Because undifferentiated SH-SY5Y cells express the functional GHS-R1a receptor [21] and differentiated SH-SY5Y cells express the G protein-coupled receptors (GPCRs) [22,23], the actions of teaghrelin on MPP^+^-induced mitochondrial dysfunction may be mediated by an off-target effect. Teaghrelin (TG) is an acylated flavonoid tetraglycoside extracted from Chin-Shin Oolong tea. Some previous studies showed that flavonoids had many benefits in neuroprotection through other receptors, ion channel, tyrosine kinase receptor [37]. However, the exact mechanisms and discrepancy should be verified by additional investigations.

In general, apoptosis is initiated after mitochondrial damage; this causes the release of cytochrome c and activation of caspase-3. In this study, we demonstrated that teaghrelin effectively alleviates MPP^+^-induced SH-SY5Y cell apoptosis. A previous study reported that ghrelin activated ERK1/2 against MPP^+^-induced apoptosis [16]. In the present study, ERK1/2 phosphorylation dramatically decreased, and cytochrome c and cleaved caspase-3 levels significantly increased after MPP^+^ treatment. Teaghrelin improved the effect of MPP^+^, but teaghrelin treatment with substance P mitigated the protective effect of teaghrelin. These results suggest that teaghrelin attenuates MPP^+^-induced apoptosis through activation of the GHS-R1a/GPCRs–ERK1/2 pathway.

Mitochondria biogenesis is a key process in the maintenance of mitochondrial mass and facilitates the replacement of damaged mitochondria for the preservation of mitochondrial function [31]. PGC-1α is a central regulator of mitochondrial biogenesis through AMPK/SIRT1 activity [28]. Increasing AMPK activity has been regarded as a therapeutic strategy for PD, which could prevent neuronal cell loss in the MPP^+^-induced PD model. Activation of the AMPK pathway could stimulate SIRT1 to deacetylate PGC-1α, subsequently facilitating mitochondrial biogenesis [31]. In the MPP^+^-induced PD model in this study, only teaghrelin treatment without substance P activated the AMPK/SIRT1/PGC-1α pathway. Similar to the effect of ghrelin, our results indicate that teaghrelin plays a crucial role in replacing damaged mitochondria by increase mitochondria biogenesis.

To date, research has revealed that the loss of DA in the striatum is the main cause of abnormal motor function in PD. The key part of DA biosynthesis is the rate-limiting step involving the formation of L-DOPA, which is a precursor molecule of DA, controlled by TH. In addition to the loss of DA, TH deficiency is thought to be a hallmark of PD [38]. However, decreased levels of DA, resulting from TH deficiency, do not play a direct role in cell survival [39]. In a PD mouse model, DA concentration and TH expression in the striatum were reported to be increased by ghrelin [40]. In the present study, the immunoflouresence results showed that MPP^+^ treatment significantly induced cell death. With teaghrelin treatment in the MPP^+^ group, cell counts and TH activity significantly increased. Furthermore, the quantification of TH activity through Western blotting indicated that teaghrelin restored the loss in TH activity.

We previously demonstrated that teaghrelin, a ghrelin analog extracted from Chin-Shin Oolong tea, effectively prevents muscle atrophy [28]. In the present study, we found that teaghrelin activated the AMPK/SIRT1/PGC-1α and ERK1/2 pathways to antagonize MPP^+^-induced cell death. In conclusion, teaghrelin is a potential candidate for the therapeutic treatment of PD.
